# A Dual Cell‐Line Molecular Platform to Assess Sub‐Threshold Biocompatibility: Application to Processed Orthodontic Clears Aligners

**DOI:** 10.1111/ocr.70119

**Published:** 2026-04-07

**Authors:** Katia Barbaro, Ettore Candida, Francesca Silvestrini Biavati, Valentina Lanteri, Paola Ghisellini, Cristina Rando, Roberto Eggenhöffner, Alessandro Ugolini

**Affiliations:** ^1^ Istituto Zooprofilattico Sperimentale Del Lazio e Della Toscana “M. Aleandri” Rome Katia Barbaro Italy; ^2^ Department of Surgical Sciences and Integrated Diagnostics (DISC) Genova University Genoa Italy; ^3^ Surgical, Medical and Dental Department University of Modena and Reggio Emilia Modena Italy

**Keywords:** biocompatibility, hormone receptors, inflammatory markers, orthodontic clear aligners, polyethylene terephthalate glycol‐modified (PETG)

## Abstract

**Objective:**

Traditional biocompatibility assessments primarily address cytotoxicity and bulk material properties but often overlook molecular responses that occur below toxicity thresholds. Detecting these subtle effects is crucial for materials intended for prolonged contact with human tissues. This study uses a dual‐cell model using MCF‐7 and THP‐1 lines to evaluate low‐level hormonal and immune responses. In this context, clear aligners—removable thermoplastic devices increasingly used in orthodontics—require careful evaluation due to their extended intraoral use and design‐dependent clinical performance.

**Materials and Methods:**

Cell orthodontic aligners manufactured with CNC milling or laser cutting served as test samples. Cell viability and cytotoxicity were monitored using the MTT assay for up to 7 days, while the evaluation of early gene expression response was carried out with RT‐PCR. The molecular endpoints included inflammatory markers and hormone sensitive receptors (ERα, ERβ). Gene expression was detected on known inflammatory and hormone‐inducible genes (COX‐2, IL‐6, IL‐8, ERα/ERβ), selected as representative indicators of inflammatory and hormone signalling, while other cytokines were also measured to provide more detail to sensitivity profiling.

**Results:**

Despite preserved viability, modest modulation of COX‐2 was detected in THP‐1 cell lines in response to milled orthodontic clear aligners, without concomitant induction of IL‐6 or IL‐8. The expression of ERα/ERβ did not change under any of the conditions. All the responses remained significantly lower than the EC10 levels established for xenoestrogens and inflammatory mediators, thereby verifying absence of biologically adverse or pathogenic activation of the material.

**Conclusion:**

THP‐1 and MCF‐7 pair is a bioactivity detection sub‐threshold system that is both efficient and sensitive as well as a highly effective preclinical test for dental material. The findings of this study confirm that orthodontic clear aligners, even after being trimmed on 3D‐printed models, do not exhibit antigenic properties. This supports their continued use in intraoral applications where the absence of immunological reactivity is essential during the development phase.

## 1 Introduction

Clear aligner therapy is a common alternative to conventional fixed treatments in orthodontics. Since the planning of the orthodontic treatment with clear aligners is made by specific software, the introduction of digital technologies in dentistry gave a great impulse in the use of removable aligners [1]. Nowadays, different companies from many countries produce their own type of clear aligners [2]. The main difference between fixed appliances and clear aligners is that the latter can be removed from the mouth; however, to obtain satisfying results, every aligner has to be worn for up to 22 h/day for 7–14 days, according to the manufacturer's protocol [3]. According to the complexity of the case, the number of aligners is different for each patient. Clear aligners can be especially made of polyethylene terephthalate (PET) and glycol‐modified polyethylene terephthalate (PETG). The stability and mechanical properties make these thermoplastic materials suitable for the intraoral environment [4]. Usually, the most common method of aligner manufacturing involves deep drawing of a thermoplastic sheet onto a 3D‐printed model [5]. After that, the aligner can be cut on the 3D‐printed model with different methods. This step, also, could affect the safety of the aligner. Modern biomaterials, particularly those intended for implantation in living tissues, require evaluation beyond simple toxicity testing [6, 7]. They need to be assessed for how they interact with the molecular processing governing cell action. Conventional methods of assessing in vitro testing such as cell necrosis or tissue morphology may overlook more subtle responses which could alter how the biomaterial integrates with tissue, incite inflammation, or result in long‐term complications [8, 9]. The need for more sensitive, mechanism‐informed screening platforms has grown alongside the use of polymeric materials in medical applications, particularly those that undergo machining, shaping, or surface treatment [10, 11]. These mechanisms have the ability to produce complex changes on the surface or create molecules that, although non‐toxic for cellular integrity, can affect gene function, be recognized by the immune system, or mimic hormonal signals.

In this context, using molecularly responsive in vitro models provides a viable answer [9]. Specifically, the use of MCF‐7 human epithelial cells allows the investigation of the estrogenic effects of different compounds [12]. In parallel, THP‐1 human monocytic cells serve as a representative model of immune responses [9]. Together, these two cellular systems offer complementary perspectives on how substances interact with distinct cellular pathways. These two cell lines are genetically stable and well‐characterized and are sensitive to various agents of both chemicals and physical properties. Their importance is their ability to enable quantitation of early‐response genes, such as canonical markers COX‐2, IL‐6, IL‐8, and ERα/β, through changes that do not approach toxic levels.

The present study exploits and proves an effective, non‐invasive, and scalable dual cell‐line molecular framework for the evaluation of biocompatibility in the preclinical environment. By applying this platform to polyethylene terephthalate glycol‐modified custom hybrid copolyester (PETG‐CHC) samples subjected to different mechanical processing techniques, we demonstrate how early transcriptional responses, despite an absence of cytotoxicity, can reveal meaningful distinctions in cellular reactivity [13]. Moreover, we position this approach as a generalizable strategy for materials screening, bridging the gap between conventional assays and next‐generation biocompatibility evaluation.

## 2 Materials and Methods

### 2.1 Conceptual Design of the Experimental Platform

**FIGURE 1:**
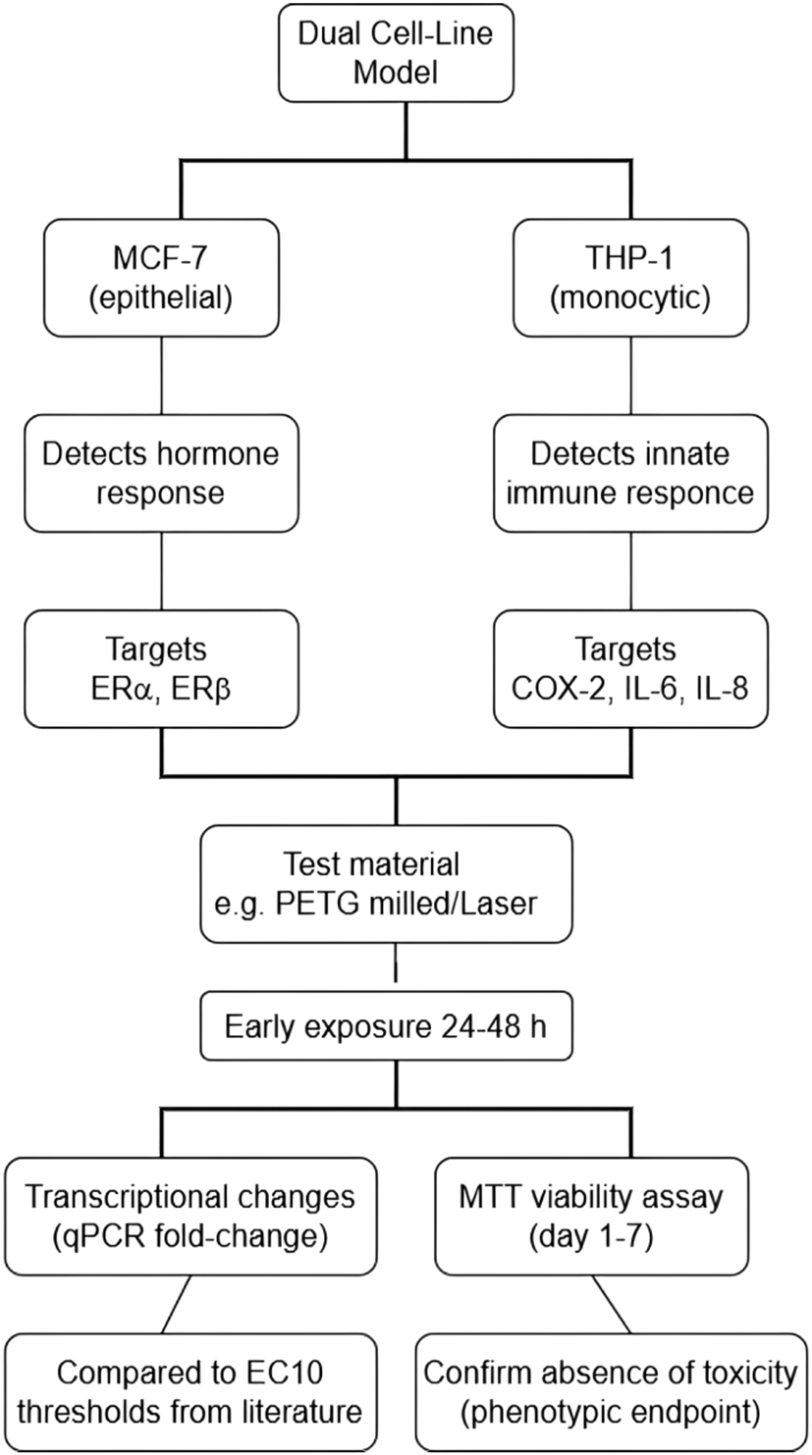
Schematic overview of the dual cell‐line molecular platform used to evaluate early, sub‐toxic biological responses to candidate biomaterials. Gene expression changes after 24–48 h exposure are compared to EC10 thresholds derived from literature, enabling detection of molecular reactivity even in the absence of cytotoxicity, which is assessed in parallel by MTT assay.

The present study was designed to evaluate the capacity of a dual cell‐line system to detect early and sub‐threshold biological responses to biomaterial exposure. As illustrated in Figure 1, the system combines two unique cell lines of human origin that respond with differing yet complementary reactions to various classes of stimulants. The MCF‐7 cell line, derived from human breast epithelial tissue, is oestrogen receptor‐positive and is used mainly for the testing of hormonal activity, such as the screening of endocrine‐disrupting chemicals [14, 15, 16, 17, 18]. The THP‐1 cell line, which is of monocytic origin, is more sensitive to immune and pro‐inflammatory impulses and is often used for the characterization of innate reactions to xenobiotics and particulate matter [19].

Dual‐cell methodology enables the detection of a broad spectrum of biologically relevant signals. MCF‐7 cells allow for important information regarding oestrogen or stress‐induced responses generated from interactions with the adjacent tissue, while THP‐1 cells track sudden pro‐inflammatory responses. MCF‐7 cells were selected to detect potential estrogenic or endocrine‐disrupting responses to polymer residual products, while THP‐1 cells capture inflammatory signalling.

Combined, these cell lines form a powerful in vitro system that can define subtleties of cellular disruptions even in the absence of a clear cytotoxicity effect.

In this context, PETG‐CHC samples were selected not as novel test materials, but as a well‐characterized reference substrate to challenge the system's sensitivity under clinically relevant conditions. Although PETG‐CHC is widely regarded as biocompatible, its inclusion allowed us to investigate whether refined surface modifications—such as laser cutting or mechanical milling—might elicit low‐grade, sub‐toxic cellular responses detectable by this dual‐line platform [20].

An overview of the dual cell‐line system used to check early low‐level biological responses to potential biomaterials is schematized in Figure 1. MCF‐7 cells track activation of estrogenic pathways through ERα and ERβ, while THP‐1 cells react to immune signals by producing COX‐2, IL‐6, and IL‐8. Changes in gene expression after 24 h of exposure are compared to EC10 thresholds from the literature, allowing us to spot molecular reactions even when there's no cell damage, which we also check using the MTT assay. The PETG‐CHC samples used in this study served not as a test material per se, but as a well‐characterized reference to challenge the system's sensitivity under real‐world conditions. Although PETG‐CHC is broadly recognized as biocompatible, its inclusion here allowed us to assess the platform's responsiveness to subtle, sub‐toxic effects potentially induced by clinically relevant surface modifications.

In this study, THP‐1 cells were intentionally used in their undifferentiated, monocytic state to detect subtle immune signalling changes. Unlike PMA‐differentiated into macrophage‐like phenotypes the undifferentiated THP‐1 model retains a high responsiveness to low‐level stressors and avoids saturation of transcriptional responses [21]. Thus, this approach was chosen to maximize the platform's sensitivity towards subtle, sub‐toxic molecular shifts while avoiding the high transcriptional ‘noise’ and pre‐activation of inflammatory pathways typically induced by differentiating agents. By utilizing non‐differentiated monocytes, the model acts as a highly sensitive ‘sentinel’ system capable of capturing the very first stages of cell‐material interaction and early molecular adaptation. Combined, these cell lines form a powerful in vitro system that can define subtleties of cellular disruptions even in the absence of a clear cytotoxicity effect.

### 2.2 Experimental Samples and Exposure Setup

The thermoplastic material used in this study is a hybrid copolyester of PET‐G [Nuvola Essential, Linea Giove, G.E.O. S.r.l. (Gruppo Europeo di Ortodonzia) Via del Progresso 14–00030 San Cesareo (RM)]. The thermoplastic material was cut on the 3D‐printed model with a milling cutter [Tungsten Carbide Drill coated with DLC] or CO_2_ laser cutting [Infinity Plus, Iradion Laser Inc. Uxbridge, MA 01569–2235, USA].

Circular samples of PETG‐CHC were produced using two common surface processing methods: CNC milling (10 samples) and CO_2_ laser cutting (10 samples). These processing techniques represent most typical manufacturing practices used in biomedical device prototyping and patient applications. The resulting samples had uniform dimensions (10 mm diameter, 1 mm thickness) and were carefully washed with phosphate‐buffered saline (PBS) to remove debris or processing residues before cellular exposure.

For each experiment, cells were cultured in standard 24‐well plates and allowed to reach appropriate confluence before the introduction of the PETG‐CHC samples. One sample was carefully placed in direct contact with the suspended or adherent cellular layer. MCF‐7 cells were cultured in DMEM with addition of 10% foetal calf serum and antibiotics with an initial seeding density of 1 × 10^5^ cells per well, while THP‐1 cells were cultured in RPMI‐1640 with similar supplements with an initial seeding density of 2 × 10^5^ cells per well. All cell cultures were maintained in a humidified atmosphere with 5% CO_2_ and a temperature of 37°C. The discs were sterilized in 70% ethyl alcohol for 30 min and washed 3 times in sterile PBS.

Exposure times were set to seek out initial molecular events: gene expression analysis was performed after 24 and 48 h, while viability and cytotoxicity measurements reached up to 7 days. The negative controls consisted of control wells with cells not exposed to PETG‐CHC; this research did, however, not utilize any positive controls since it aimed for comparison with reported EC10/EC50 values from the literature instead.

### 2.3 RNA Isolation and Relative Assessment of Gene Expression

Following exposure for the duration of 24 to 48 h, total RNA was extracted from the cells using commercially procured silica column purification (Qiagen RNeasy Mini Kit, Qiagen, Germany), according to the procedural protocols supplied with the kit. The amount and the purity of RNA were assessed through spectrophotometric measurements (NanoDrop ND‐1000, Thermo Fisher Scientific), with resulting A260/A280 ratio values between approximately 1.8 and 2.0, indicative of high RNA purity.

One microgram of total RNA per sample was used for the synthesis of cDNA using validated reverse transcription kit with controlled thermal cycling conditions. Real‐time quantitative polymerase chain reaction (qPCR) was performed using SYBR Green chemistry on a calibrated 96‐well thermal cycler on a CFX96 Real‐Time PCR Detection System (Bio‐Rad Laboratories). The mixture for each reaction included template cDNA, validated gene‐specific primers chosen for efficiency and specificity, and master mix components (the list of the qPCR primers for the examined genes is reported in Section S1). The amplification cycle used optimized steps designed for the target genes, including denaturation, annealing, and extension processes. In particular, COX‐2 (PTGS2), IL‐6, and IL‐8 were used as known markers of inflammation and cellular stress, along with ERα (ESR1) and ERβ (ESR2) to evaluate possible estrogenic activity.

GAPDH was used as the internal housekeeping gene for the normalization process [22]. GAPDH was used as the internal reference gene for normalization. This gene was selected based on its widespread use and documented expression stability in both MCF‐7 and THP‐1 cells under non‐cytotoxic experimental conditions. In the present study, GAPDH Ct values showed minimal variation across treatments and time points, supporting its suitability as a housekeeping gene.

The levels of each target gene were validated using the comparative Ct (2^−ΔΔCt^) method, with untreated cells used as the standard control reference. Each experimental condition was measured in biological triplicates, and each PCR reaction in technical duplicates. Melt curve analysis was systematically performed to confirm amplification specificity and exclude primer‐dimer or non‐specific products.

### 2.4 Cell Viability Assay

MTT test was used to measure the cell viability to compare cellular metabolic function for the different treatment regimens. MCF‐7 cells and THP‐1 cells were exposed for 24 h, 48 h, 5 days, or 7 days with PETG‐CHC samples. At each time point, the culture medium was replaced with fresh medium containing 0.5 mg/mL MTT (Sigma‐Aldrich, M5655). Plates were incubated at 37 C for 4 h to allow formazan formation by viable cells [23].

Following incubation, the supernatant was carefully aspirated, and the formazan crystals were dissolved in 100 μL of DMSO. Absorbance was measured at 570 nm using a microplate reader (Bio‐Rad iMark). Values were measured against control cells that did not undergo treatment. Each experimental condition was tested in technical triplicate and performed in three independent biological replicates.

### 2.5 Sensitivity Benchmarking and Interpretation Framework

To place gene expression changes into biological perspective, measured fold change levels were matched against published levels of EC10 and EC50 levels for the same cell type and for molecular endpoint. In MCF‐7 cells, for example, transcription induction of the oestrogen receptor alpha (ERα) by 17β‐estradiol has ubiquitously been observed across concentrations from 10^−12^ to 10^−9^ mol/L, with biological response typically requiring two‐ or three‐fold induction [24, 25]. In parallel, THP‐1 cell experiments have demonstrated that COX‐2 and IL‐6 expression can be induced via inflammation‐related mechanisms. This induction has been reported following exposure to bacterial lipopolysaccharide (LPS) [26]. Similar responses have also been observed with engineered nanoparticles, typically at nanomolar to low micromolar concentrations. In these conditions, fold inductions of three or greater are commonly reported. Additional interleukin targets such as IL‐1B and IL‐10 were included in the primer sets as exploratory sensitivity extensions to expand the sensitivity profile of the THP‐1 inflammatory response. These were assessed using the same qPCR protocols described, though they were not emphasized in the original gene panel.

To further contextualize the transcriptional data, we computed an Inflammatory Balance Index (IBI) for THP‐1 responses, defined as the ratio of the sum of pro‐inflammatory mediators (COX‐2 and IL‐6) to the modulatory cytokine IL‐8. As reported in detail in the Section S2, this derived metric provides a further, simplified, dimensionless indicator of signal balance, useful for capturing shifts in inflammatory tone.

By applying both defined biological thresholds and integrative indices such as IBI, the transcriptional reactions described in this study can be evaluated statistically as well as in terms of variation regarding defined levels of activation linked with functional cellular responses. This benchmarking approach allows a more refined evaluation of system responsiveness. It enables the detection of low‐threshold molecular reactions that occur independently of cytotoxicity. These reactions may indicate early‐stage biological responsiveness. Importantly, they do not necessarily arise from overt cytotoxic effects or from clearly observable phenotypic features. The ability to detect such modest but reproducible transcriptional shifts supports the value of the dual‐cell platform as a sensitive tool for screening biomaterials at the interface of safety and bioactivity.

### 2.6 Statistical Analyses

Statistical analyses of gene expression values were calculated using the 2−^ΔΔCt^ method with the GraphPad Prism (version 9.5, GraphPad Software, San Diego, CA). Where applicable, results were analysed by one‐way ANOVA, followed by Tukey's post hoc test for multiple comparisons. For MTT viability data, standard deviations were calculated from biological triplicates, and ANOVA was applied for comparisons across timepoints and treatment groups. A *p*‐value < 0.05 was considered statistically significant. In figures, error bars represent propagated errors from a conservative estimation of the global experimental errors that turn out to be larger than statistical values. The method allows conservative yet interpretable quantification of sub‐threshold molecular changes. More details are reported in the Section S5.

## 3 Results

### 3.1 Platform Responsiveness to a Non‐Toxic Material

To assess whether the proposed dual cell‐line platform can detect early molecular perturbations below the cytotoxic threshold, we used PETG‐CHC samples processed by CNC milling or CO_2_ laser cutting as a case study. PETG‐CHC is known to be biologically inert at the cellular level, and previous studies, including our own chemical analysis, confirmed the absence of leachable compounds such as bisphenol A (BPA), phthalates, or residual solvents [27].

Despite this chemical inertness, distinct transcriptional responses were observed. In THP‐1 cells, modulation of COX‐2 transcription was detected following exposure to milled PETG‐CHC, with log₂ fold change values indicating a modest deviation from baseline, while IL‐6 and IL‐8 levels remained close to control values. In the MCF‐7 cell line, there was mild augmentation of ERα with the same treatment, with evidence of the log₂ fold change being approximately 0.56, while ERβ showed minimal changes in transcription levels.

All experimental conditions maintained cell viability levels of 85% or higher, indicating that cytotoxicity could not account for the observed transcriptional effects. No alterations in cell morphology or proliferation were detected during the exposure window. This pattern suggests a localized molecular adaptation at the cell–material interface rather than the initiation of an overt inflammatory or stress response. By contrast, PETG‐CHC samples processed by laser cutting produced only minimal changes in gene expression across both cell models, reinforcing their biologically neutralbehaviour. Collectively, these findings confirm the platform's capacity to detect sub‐threshold molecular activity in the absence of phenotypic alterations.

### 3.2 Specificity of Molecular Responses

A critical requirement for any in vitro biocompatibility platform is its specificity, i.e., the ability to detect relevant cellular responses without triggering unrelated or systemic signals. In this context, Figure 3 illustrates the expression profiles of inflammatory markers in THP‐1 cells after 24 and 48 h of exposure to PETG‐CHC samples processed by either milling or laser cutting.

COX‐2 expression showed consistent transcriptional modulation following exposure to milled PETG‐CHC, with fold‐change values consistently below control levels at both 24 and 48 h. By contrast, IL‐6 and IL‐8 exhibited only minor fluctuations under all test conditions and time points, with values generally remaining within ±10%–15% of controls. Laser‐treated PETG‐CHC induced minimal changes across all genes, suggesting that this surface finishing process does not provoke a measurable biological response in these inflammatory markers.

This pattern reinforces the biological specificity of the platform, with COX‐2 stress responding selectively to surface processing, while the absence of coordinated IL‐6 and IL‐8 modulation confirms the lack of a broader inflammatory cascade.

The consistent behaviour of COX‐2 in the absence of IL‐6 or IL‐8 induction supports the interpretation of a localized, pathway‐specific response rather than generalized cellular stress. These results suggest that the platform can distinguish between innocuous and stress‐inducing surface modifications without producing non‐specific or systemic transcriptional noise. The observed expression changes remained below EC10 thresholds for inflammatory mediators, reinforcing the conclusion that processed PETG‐CHC does not pose a pro‐inflammatory risk under these test conditions, consistent with a sub‐toxic, non‐antigenic response to the tested materials.

In addition to the core markers (COX‐2, IL‐6, IL‐8) previously discussed, further evaluation of interleukin gene expression revealed detectable transcriptional activity for IL‐1B and IL‐10, primarily in the THP‐1 cell line following exposure to processed PETG‐CHC. Notably, IL‐1B showed a modest but reproducible transcriptional signal in response to milled PETG‐CHC, with CT media values clustering around 34.4–35.5, consistent with late amplification. IL‐10 was also sporadically detected under the same conditions, with CT values typically exceeding 37, indicating marginal transcriptional activation.

In contrast, laser‐treated PETG‐CHC samples yielded lower transcriptional signals for both IL‐1B and IL‐10, with CT values ranging from 34 to 38. These results reflected minimal cytokine expression, generally comparable to or slightly above background levels observed in controls. No substantial modulation of IL‐8 was observed under any processing condition.

These findings, although modest in magnitude, reinforce the platform's ability to detect fine‐scale immunological shifts that fall below overt inflammatory thresholds. The inclusion of additional interleukin markers such as IL‐1B and IL‐10 expands the interpretative range of the dual‐cell system, capturing subtle signatures of cell–material interaction that might be overlooked by standard markers alone. All interleukin‐related transcriptional changes remained below EC10‐equivalent activation levels and were unaccompanied by viability loss or phenotypic alterations, thereby confirming the sub‐toxic and non‐antigenic character of both milled and laser‐treated PETG‐CHC samples.

### 3.3 Sensitivity Compared to EC10–EC50 Thresholds

To determine whether the molecular signals detected fall within biologically significant ranges, we compared the fold changes obtained with those known to be triggered by well‐characterized positive controls (literature EC10/EC50). For example, in MCF‐7 cells, ERα activation by 17β‐estradiol occurs at 10^−12^ to 10^−9^ M concentrations, typically producing fold changes > 3.0. COX‐2 and IL‐6 in THP‐1 are upregulated by LPS or metal nanoparticles at similar nanomolar‐to‐micromolar EC10 levels, again yielding multi‐fold induction.

As illustrated in Figure 4, the fold‐change values observed for milled PETG‐CHC represent the highest transcriptional responses detected under the present experimental conditions and therefore provide a conservative basis for comparison with EC10 thresholds. In contrast, the changes observed with milled PETG‐CHC exposure remained consistently below EC10‐equivalent fold change thresholds at all evaluated time points (1.5–1.8 vs. EC10 = 2–3), indicating that the material did not elicit a biologically active response, while the platform was sufficiently sensitive to detect sub‐threshold changes. For clarity, extended exposure up to seven days applies to ERα and ERβ in MCF‐7 cells, whereas inflammatory markers in THP‐1 cells were assessed at 24 and 48 h.

These results position the system as a sentinel tool capable of identifying transcriptional shifts in the early phases of cell–material interaction, even in the absence of phenotypic outcomes.

### 3.4 Integration With Phenotypic Viability Data

While not the primary endpoint of the present study, MTT assays confirmed that the observed molecular responses were not associated with reduced cell viability. All experimental conditions maintained viability above 80% of control values, and both MCF‐7 and THP‐1 proliferated normally in the presence of PETG‐CHC, regardless of the processing method. In MCF‐7 cells, transcriptional modulation of ERα was observed, but the magnitude of the response remained below thresholds associated with functional estrogenic activation.

To further contextualize these transcriptional changes, the IBI index was calculated as reported in the Section S2, incorporating fold‐change values for COX‐2, IL‐6, and IL‐8. Milling conditions yielded elevated IBI values at both 24 h and 48 h, whereas laser‐treated samples consistently displayed IBI values close to unity.

Importantly, the elevated IBI values observed under milling conditions did not reflect coordinated pro‐inflammatory activation but instead arose primarily from marked suppression of IL‐8 expression in the absence of concomitant induction of IL‐6 or COX‐2. In contrast, laser‐treated samples showed balanced expression across all markers, consistent with molecular homeostasis. Table 1 provides the numerical synthesis of the THP‐1 inflammatory balance analysis and complements the gene‐specific data by integrating COX‐2, IL‐6, and IL‐8 into a single dimensionless descriptor. As shown in Table 1, milling generated persistently high IBI values at both time points, whereas laser‐treated samples displayed low and stable values, supporting the interpretation of a more biologically neutral profile.

**TABLE 1:** Inflammatory Balance Index (IBI) values for THP‐1 cells after 24 h and 48 h exposure to milled or laser‐processed PETG‐CHC, calculated from fold‐change values of COX‐2, IL‐6, and IL‐8.

Processing	Time	FC (COX‐2)	FC (IL‐6)	FC (IL‐8)	IBI
Milling	24 h	0.508	1.138	0.142	**11.6**
Milling	48 h	0.432	1.081	0.135	**11.2**
Laser	24 h	0.607	1.134	1.224	**1.43**
Laser	48 h	0.516	1.077	1.162	**1.37**

This dissociation between preserved cell viability and transcriptional modulation highlights the added value of molecular profiling assays, which can detect early, sub‐threshold biological responses that may not be apparent in phenotype‐based assays alone. Accordingly, the dual‐cell platform enhances the interpretive resolution of in vitro biocompatibility testing by integrating viability outcomes with pathway‐level molecular information.

## 4 Discussion

The ability to detect early and subtle biological responses to biomaterials remains a key challenge in current biocompatibility testing. Traditional cytotoxicity assays often overlook molecular alterations that precede phenotypic changes, limiting their predictive power—especially when evaluating high‐performance or surface‐modified materials. The dual‐cell platform presented here addresses this gap by providing sensitive, pathway‐specific readouts of sub‐toxic cellular responses.

As outlined in Figure 1, the system integrates two distinct human cell lines—MCF‐7 (epithelial, oestrogen‐sensitive) and THP‐1 (monocytic, inflammation‐sensitive)—to capture a broader spectrum of biologically relevant responses. This dual configuration is designed to minimize model‐specific bias and improve the resolution of early molecular effects at the cell–material interface.

**FIGURE 2:**
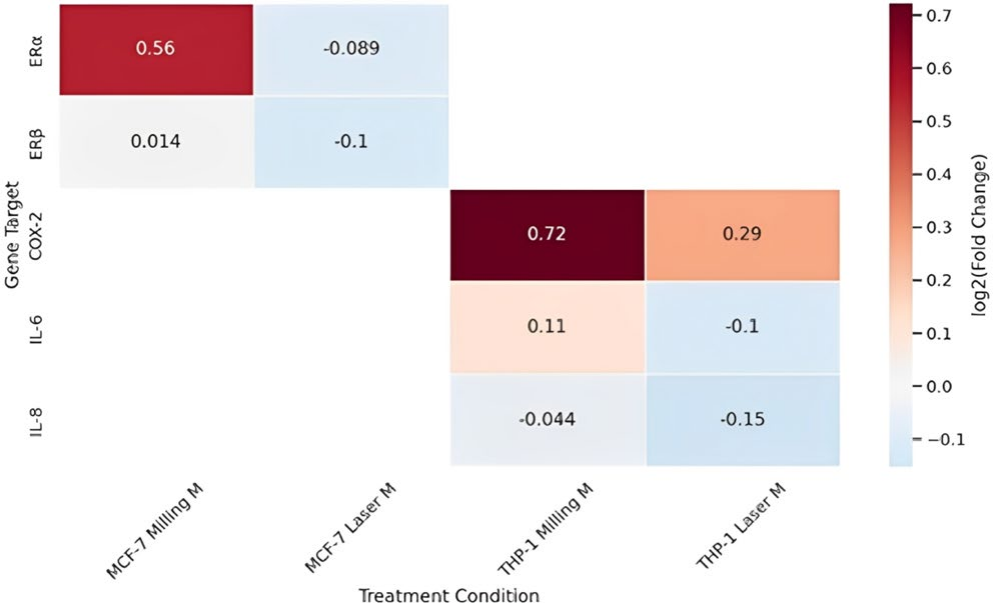
Schematic overview of the dual cell‐line molecular platform for early biocompatibility screening, showing log₂ fold change in gene expression after 24‐h exposure to PETG‐CHC samples. MCF‐7 cells were analysed for ERα and ERβ, while THP‐1 cells were assessed for COX‐2, IL‐6, and IL‐8. Modest transcriptional modulation was observed primarily for ERα in MCF‐7 cells and for COX‐2 in THP‐1 cells following milling, whereas laser‐processed samples induced minimal changes across all targets.

The platform's specificity is demonstrated in the heatmap (Figure 2), which shows selective transcriptional modulation of COX‐2 and ERα in response to milled PETG‐CHC. Crucially, IL‐6, IL‐8, and ERβ remained near baseline, supporting the interpretation of a localized, non‐inflammatory response. These results suggest that the transcriptional modulation is pathway‐specific rather than indicative of generalized cellular stress, reducing the risk of false‐positive outcomes in compatibility assessment.

**FIGURE 3:**
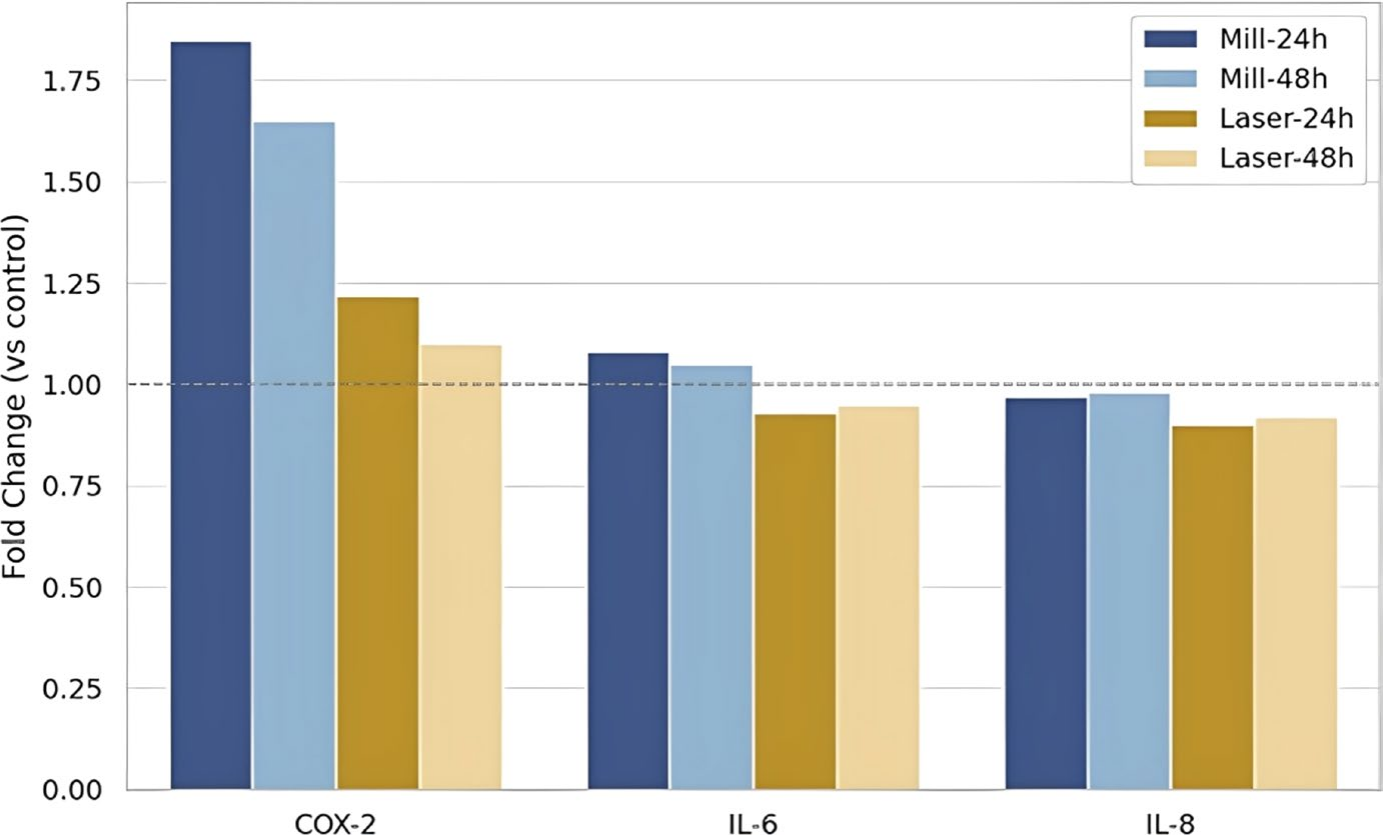
Fold‐change expression of target genes in THP‐1 cells after 24/48‐h exposure to processed PETG‐CHC as stated in the inset of the figure. Data represents mean of *n* = 3 biological replicates; error bars denote ±13% propagated from experimental uncertainties as discussed in the Section S3, numerical details on Gene Expression and Inflammatory Balance Index (IBI) in the Section S4.

The temporal dynamics of these responses are detailed in Figure 3. In THP‐1 cells, COX‐2 showed reproducible transcriptional modulation at 24 h and remained comparably modulated at 48 h, whereas IL‐6 levels remained close to baseline and IL‐8 expression exhibited condition‐dependent suppression. These patterns reinforce the hypothesis that surface‐processed PETG‐CHC induces early transcriptional adjustments without triggering an inflammatory cascade. The absence of concomitant induction of IL‐6 and IL‐8 further supports the conclusion that COX‐2 modulation reflects adaptive signalling rather than immune activation.

To further contextualize these transcriptional variations, Inflammatory Balance Index (IBI) values were calculated using fold‐change data from THP‐1 cells. Milled PETG‐CHC yielded elevated IBI values driven primarily by IL‐8 suppression, whereas laser‐treated samples consistently exhibited IBI values close to unity, indicative of molecular homeostasis. These findings point to localized molecular sensing in response to surface processing rather than to a complete inflammatory response induced by PETG‐CHC. This divergence is further supported by Table 1, in which the IBI remained markedly elevated under milling at both 24 h and 48 h (11.6 and 11.2, respectively), whereas laser‐treated samples maintained values close to unity (1.43 and 1.37). In interpretative terms, this numerical separation reinforces the view that milling is associated with an unbalanced transcriptional profile driven mainly by IL‐8 suppression, while laser processing preserves a substantially homeostatic inflammatory signature.

Exploratory co‐modulation networks presented in Section S2 are intended to support qualitative interpretation of gene‐level relationships and are not used for formal quantitative inference.

**FIGURE 4:**
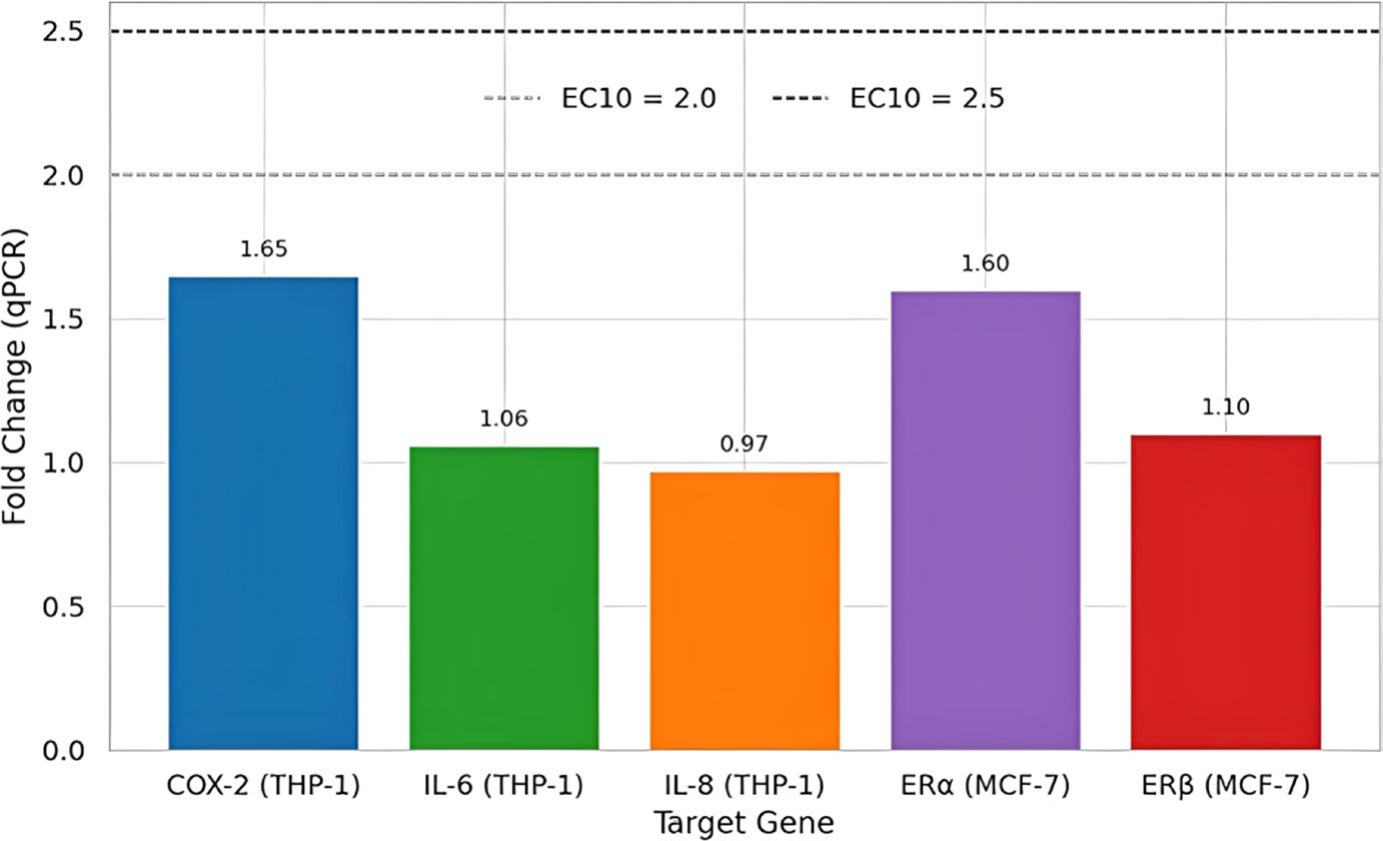
Comparison of observed gene expression levels with established EC10 thresholds. Fold changes in gene expression following exposure to milled PETG‐CHC are shown alongside literature‐derived EC10 thresholds for the same markers in MCF‐7 and THP‐1 cells. None of the observed values exceed these sensitivity thresholds, confirming the material's molecular neutrality while validating the platform's ability to detect low‐level biological changes below toxic limits. Extended exposure up to seven days applies to ERα and ERβ in MCF‐7 cells, whereas inflammatory markers in THP‐1 cells were assessed at 48 h. All values represent average expression from *n* = 3 biological replicates; error bars denote ±13% propagated from experimental uncertainties as discussed in the Section S3.

In Figure 4, these results are reported in contrast to the higher EC10 benchmarks derived from the literature. The fold changes observed +1 remain below the activation thresholds typically associated with well‐characterized stimuli such as LPS or 17β‐estradiol. This confirms that while the platform is sensitive to early transcriptional shifts, these responses remain well within sub‐threshold, non‐adverse ranges. Additional interleukin targets (IL‐1B, IL‐10) further supported the system's sensitivity to low‐level immune modulation. Milled PETG‐CHC was associated with detectable IL‐1B transcription and sporadic IL‐10 expression in THP‐1 cells, whereas laser‐treated samples showed minimal or absent cytokine signals. These differences are consistent with the platform's ability to resolve fine variations in immunological engagement without cytotoxicity.

Taken together, the agreement between molecular data, phenotypic outcomes, and literature‐derived thresholds supports a coherent, multidimensional assessment of biocompatibility. The platform detects subtle but specific molecular modulation without conflating it with toxicity, thereby improving the resolution and reliability of in vitro testing.

Finally, the comparison between PETG‐CHC processing methods indicates a more neutral biological profile for laser treatment relative to milling. Although milled PETG‐CHC was associated with more pronounced transcriptional modulation, these effects remained sub‐threshold and were not accompanied by adverse viability outcomes. Laser‐cut PETG‐CHC, by contrast, produced minimal transcriptional and phenotypic changes, supporting its suitability for applications requiring biologically neutral interfaces.

## 5 Conclusions

This study demonstrates that a dual cell‐line molecular platform—based on MCF‐7 and THP‐1 human cell models—can sensitively and specifically detect early transcriptional responses to biomaterials, even in the absence of cytotoxicity or explicit phenotypic effects. By applying this platform to a well‐characterized, non‐toxic material such as PETG‐CHC, we showed that localized cellular responses can be detected below classical activation thresholds, validating the system's ability to function within the sub‐toxic biological range.

The platform showed gene‐ and pathway‐specific responsiveness, with detectable, pathway‐resolved molecular responses to surface‐induced signals, particularly involving COX‐2 and ERα, without evidence of broader inflammatory or endocrine pathway activation. Importantly, it revealed minor, contact‐associated molecular activity without triggering broader inflammatory or endocrine pathways, confirming both the safety of the test material and the discrimination power of the model. When integrated with viability data and benchmarked against known EC10–EC50 thresholds, this system provides a nuanced and reproducible readout of early cell–material interactions. This confirms that the observed molecular responses occurred in metabolically competent cells and were not secondary to cytotoxic damage, in agreement with MTT findings. The absence of phenotypic alteration reinforces the reliability of the platform for detecting sub‐toxic bioactivity.

We propose that this dual‐line molecular approach serves as a valuable addition to the biocompatibility assessment toolkit. It can be readily adapted to screen emerging materials, surface treatments, or composite formulations and may ultimately support preclinical risk evaluation and regulatory submissions by revealing biological responses that standard assays overlook.

## Author Contributions

Conceptualization, K.B., E.C., R.E. and A.U.; methodology, K.B., V.L., P.G., and C.R.; validation, K.B., E.C., F.S.B. and A.U; investigation, K.B., E.C. P.G., C.R. and A.U.; writing – original draft preparation, K.B., E.C., F.S.B., V.L., P.G., C.R., R.E. and A.U.; writing – review and editing K.B., E.C., F.S.B., V.L., P.G., C.R., R.E. and A.U.; visualization, K.B., E.C., R.E. and A.U.; supervision, K.B., E.C., R.E. and A.U.; project administration, A.U.

## Ethics Statement

Ethical approval was not required for this work as it was conducted entirely in vitro with no involvement of human participants or animals.

## Conflicts of Interest

The authors declare no conflicts of interest. ID 0828f12c‐fd17‐4d3f‐b60a‐f42112b91433.

## Supporting information


**Data S1:** Section S1. qPCR primers details for the examined genes.
Section S2. Inflammatory Balance Index (IBI): Definition and Example Calculations.
Section S3. Treatment and Representation of Experimental Errors.
Section S4—Statistical Analysis and Illustrative Examples.

## Data Availability

The data that support the findings of this study are available from the corresponding author upon reasonable request.
