# Supplementary material for: A Dual Cell‐Line Molecular Platform to Assess Sub‐Threshold Biocompatibility: Application to Processed Orthodontic Clears Aligners
Source: Orthod Craniofac Res. 2026 Apr 7;29(4):671–9. doi: 10.1111/ocr.70119 (PMC13485172; doi:10.1111/ocr.70119)
Supplement: Supplementary file 1 [file OCR-29-671-s001.docx]

**Supplementary Section 1. qPCR primers details for the examined genes.**

| **Gene Symbol** | **Target** | **Forward Primer (5→3)** | **Reverse Primer (5→3)** | **Amplicon Size (bp)** | **Annealing Temp (°C)** |
| --- | --- | --- | --- | --- | --- |
| **COX-2 (PTGS2)** | Inflammation | GTTCCACCCGCAGTACAGAA | AGGGCTTCAGCATAAAGCGT | 106 | 58 |
| **IL-6** | Pro-inflammatory | AGACAAAGCCACCACCCCTAA | CTCGTTCTGTGACTGCAGCTTATC | 120 | 60 |
| **IL-8** | Chemokine | GAAAACTGGGTGCAGAGGGT | AAAAAGGCAGATACCTAATGACGA | 156 | 57 |
| **IL-4** | Anti-inflammatory | CCAACCCTGGTCTGCTTACTG | TTGTAAGGTGATGTCGCACTTGT | 140 | 58 |
| **IL-10** | Anti-inflammatory | GCCTTCGGCCCAGTGAA | AGAGACCCGGTCAGCAACAA | 346 | 54 |
| **TNF-α** | Pro-inflammatory | AAAACAACCCTCAGACGCCA | CATGGTGTCCTTTCCAGGGG | 108 | 58 |
| **IFN-γ** | Pro-inflammatory | TGTCGCCAGCAGCTAAAACA | ACTGGGATGCTCTTCGACCT | 72 | 58 |
| **ERα (ESR1)** | Hormonal | CACATGAGTAACAAAGGCATGG | ATGAAGTAGAGCCCGCAGTG | 177 | 56 |
| **ERβ (ESR2)** | Hormonal | GGCATGCGAGTAACAAGGGC | GGGAGCCCTCTTTGCTTTT | 177 | 56 |
| **GAPDH** | Housekeeping | ACCAGGGCTGCTTTTAAC | ACGGTGCCATGGAATTTG | 125 | 54 |

**Supplementary Section 2. Inflammatory Balance Index (IBI): Definition and Example Calculations**

To complement the gene-by-gene transcriptional analysis presented in the main text, an Inflammatory Balance Index (IBI) was defined as a composite metric aimed at capturing the relative balance between early inflammatory sensing and downstream chemokine signaling in THP-1 cells following material exposure. Rather than quantifying absolute inflammatory activation, the IBI is intended to highlight asymmetries in pathway engagement that may occur below overt activation thresholds.

The index integrates fold-change values of key inflammatory mediators according to the following equation:

$$IBI = \frac{Fold Change (COX-2)+Fold Change (IL-6)}{Fold Change (IL-8)}$$

Only non-log-transformed fold change (FC) values derived directly from experimental ΔΔCt calculations were used.

In this framework, COX-2 and IL-6 are treated as early-response and modulatory mediators, while IL-8 is considered a proxy for downstream chemokine signaling and propagation. Consequently, elevated IBI values do not necessarily indicate inflammatory activation but may instead reflect suppression or lack of engagement of chemokine-mediated pathways.

IBI values were calculated for THP-1 cells that had been exposed to PETG-CHC, which was processed either by milling or laser cutting. The FC values used for these calculations were obtained experimentally at 24 and 48 hours, matching the time points shown in Figure 3.

Condition: THP-1 – Milling (48 h)
COX-2 FC = 0.43 - IL-6 FC = 1.08 - IL-8 FC = 0.13

$$\mathrm{IBI}_{Milling,48h}=\frac{0.43+1.08}{0.13}\approx11.2$$

Condition: THP-1 – Laser (48 h)
COX-2 FC = 0.52 - IL-6 FC = 1.08 - IL-8 FC = 1.16

$$\mathrm{IBI}_{Laser,48h}=\frac{0.52+1.08}{1.16}\approx1.38$$

**Interpretation and Relation to Main Figures**

Figure 3 summarizes the dynamics of individual inflammatory responses in time, while Figure 4 plots individual inflammatory response levels relative to EC10 cutoffs. The balance of the integrated signaling response is reflected through the use of the IBI. The main factor contributing to high IBI levels in the milling environment is the marked suppression of IL-8 expression relative to control levelswithout any coherent up-regulation of IL-6 and/or COX-2 expression.

Notably, all transcript expression changes related to the IBI are kept below the EC10 equivalent activation threshold, with no loss of cell viability or phenotype. In the laser-treated samples of PETG-CHC, the IBI approached unity, indicating a neutral biological effect at the molecular level.

Together, this bipartite approach to representation, in which the figures are devoted to the examination of separate genes and the supplementary materials provide a combined index, provide support for the conclusion that there is a sub-threshold level of molecular sensing which does not progress to inflammation.

**Supplementary Section 3. Treatment and Representation of Experimental Errors**

In this study, key experiments are the cell viability (MTT), and the gene expression (qRT-PCR), each associated with specific sources of variability. These were addressed using both statistical tests and error propagation formulas, alongside conservative assumptions where replication was limited.

Statistical Testing:
Where n ≥ 3, one-way ANOVA and post hoc tests were conducted. These statistical comparisons supported conclusions but were not always annotated in figures for clarity in multi-gene, multi-condition panels.

This approach—combining formal ANOVA with estimated propagated error—allowed rigorous yet interpretable comparisons across molecular, metabolic, and chemical endpoints, supporting conclusions about PETG’s safety under clinical processing scenarios.

Cell Viability:
For MTT assays, standard deviation was calculated when biological triplicates were available, and statistical significance tested by one-way ANOVA. In figures, a conservative ±10% error band was applied where SD was extremely low, acknowledging typical biological variability from pipetting or metabolic fluctuation.

Gene Expression (qPCR):
qRT-PCR data were derived from the ΔΔCt method, and fold changes (2^–ΔΔCt) were reported. When ΔΔCt standard deviations were available, fold-change error propagation followed:

  δy = ln(2) × 2^–x × δx

This yields typical fold change uncertainties of 10–20%, depending on the ΔΔCt value. For instance, typically:

- ΔΔCt = 1 → FC = 0.5 → ±20.8%
- ΔΔCt = 0.3 → FC ≈ 0.81 → ±10.8%

Across conditions, the average error stabilized at ~13%, which was adopted for graphical display when replicate error was unavailable or unrealistically low due to instrument sensitivity.

**Supplementary Section 4 – Statistical Analysis and Illustrative Examples**

This section describes the statistical framework applied to the analysis of gene expression data, including software implementation, criteria for significance, and representative examples illustrating the interpretation of transcriptional modulation within a biophysical context.

All statistical analyses were performed using Python (v3.13) with the statsmodels and scipy.stats libraries. For each gene target, ΔΔCt values were calculated from triplicate biological measurements, and fold changes were derived as $2^{-\Delta\Delta Ct}$ . When biological replicates exhibited extremely low within-group variance, a conservative estimated uncertainty of ±13% was applied, based on error propagation of qPCR measurements, as detailed in Supplementary Section 3.

Comparisons across treatment groups (Control, Milled, Laser) were conducted using one-way analysis of variance (ANOVA). When a significant group effect was detected (p < 0.05), Tukey’s Honest Significant Difference (HSD) post hoc test was applied to identify pairwise differences. Statistical testing was used to support interpretation of molecular trends rather than to imply biological relevance solely on the basis of significance.

**Example 1: TNFα Expression in THP-1 Cells (24 h)**

An illustrative example of the statistical workflow is provided using TNFα expression at 24 hours in THP-1 cells. Mean ΔΔCt values, fold changes, and associated standard deviations are reported in Table Sx.

ANOVA revealed a significant treatment effect, indicating differential transcriptional modulation across conditions. Post hoc analysis showed that the milled condition differed significantly from the untreated control, whereas laser-treated samples did not exhibit statistically significant differences relative to controls. This pattern is consistent with a modest reduction in TNFα transcription under milling conditions, without evidence of a coordinated inflammatory response.

**Example 2: COX-2 Expression in THP-1 Cells (24 h)**

A second illustrative example is provided for COX-2 expression in THP-1 cells at 24 hours. Although between-group differences were statistically detectable, fold-change values remained close to control levels and below established EC10-equivalent thresholds. Importantly, COX-2 modulation occurred in the absence of concomitant induction of IL-6 or IL-8, indicating a limited and non-propagating transcriptional response.

These examples demonstrate that statistically significant differences can be detected even when transcriptional changes remain biologically modest. Accordingly, interpretation of gene expression data was based not solely on p-values, but on the combined evaluation of fold-change magnitude, pathway coherence, temporal consistency, and comparison with established activation thresholds.

Overall, this analytical approach supports the identification of early, sub-threshold transcriptional adaptations that do not progress toward overt inflammatory activation or cytotoxicity. Such fine-scale molecular sensitivity underscores the utility of qPCR-based assays, when coupled with rigorous statistical treatment, for probing early cell–material interactions beyond traditional viability-based endpoints.
